# Epidemiological survey and sequence information analysis of swine hepatitis E virus in Sichuan, China

**DOI:** 10.1017/S0950268818002893

**Published:** 2018-11-19

**Authors:** Y. Y. Li, Z. W. Xu, X. J. Li, S. Y. Gong, Y. Cai, Y. Q. Chen, Y. M. Li, Y. F. Xu, X. G. Sun, L. Zhu

**Affiliations:** 1 Veterinary Medicine College, Sichuan Agricultural University, Chengdu, Sichuan Province, China; 2 Key Laboratory of Animal Disease and Human Health of Sichuan Province, Sichuan Agricultural University, Chengdu, Sichuan Province, China

**Keywords:** Epidemiological survey, Sichuan Province, swine hepatitis E virus

## Abstract

Hepatitis E is an important zoonosis that is prevalent in China. Hepatitis E virus (HEV) is a pathogen that affects humans and animals and endangers public health in China. In this study, the detection of HEV epidemics in swine in Sichuan Province, China, was carried out by nested real-time PCR. A total of 174 stool samples and 160 bile samples from swine in Sichuan Province were examined. In addition, software was used to analyse the biological evolution of HEV. The results showed that within 2 years of swine HEV (SHEV) infection in China, SHEV was first detected in Sichuan Province. HEV was endemic in Sichuan; the positive rate for pig farms was 11.1%, and the total positive sample rate was 10.5%. The age of swine with the highest positive rate (17.9%) was 5–9 weeks. The examined swine species in order of highest to lowest HEV infection rates were Chenghua pig, Large White, Duroc, Pietrain, Landrace and Hampshire. Nucleotide and amino acid sequence analysis showed that the HEV epidemic in swine in Sichuan Province was related to genotype IV, which had the highest homology to HEV in Beijing. Sichuan strains have greater variation than Chinese representative strains, which may indicate the presence of new HEV strains.

## Introduction

Hepatitis E virus (HEV) belongs to the Hepeviridae family Hepevirus, which is a single-strand, positive, non-enveloped RNA virus. The virus particles are spherical with a diameter of 32–34 nm. The nucleocapsid has icosahedral symmetry. The genome is approximately 7.2 kb in length and consists of three open reading frames (ORFs). HEV is divided into four major genotypes [1]. HEV-I is mainly found in Asian and African countries, HEV-II is found in Mexico and Nigeria, and HEV-III is found in the USA and European countries [2]. However, recent discoveries have also shown that HEV-III exists in Japan, Korea, mainland China and Taiwan. HEV-IV is mainly found in China, Japan and Vietnam. Japan is suspected of having genotype V of HEV. Mostly types I and IV exist in China [3–5].

Hepatitis E is a zoonotic disease, and its main route of transmission is faecal–oral transmission, where infection is caused by drinking contaminated water and eating contaminated food [6–8]. Eating uncooked HEV-infected animal tissues or offal may also lead to food-borne infections [9, 10]. Mother to child transmission [11, 12] and blood transfusion [13, 14] are also important routes of transmission. HEV infection in pregnant women can cause miscarriage or death, and the mortality rate as high as 20–30%. HEV is also found in a variety of animals; it can be transmitted between humans and pigs and is a prominent pathogen in human health and the pig industry [15, 16].

The prevalent forms of HEV genotypes in China are mainly genotypes I, III and IV. Genotype I is mainly located in Xinjiang and Beijing, genotype III is located in Shanghai and the neighbouring provinces of Jiangsu, and genotype IV is the predominant genotype of HEV with a broad distribution; its main areas include Northeast, Central, Northwest and East China. However, information on HEV is still missing in some areas of China, such as Northwest China (Ningxia, Shaanxi and Qinghai), Southwest China (Sichuan, Yunnan, Guizhou, Tibet and Chongqing) and South China (Guangdong and Hainan) [17]. This article mainly studies the prevalence of HEV in Sichuan Province and lays the foundation for research on the prevalence of HEV in China.

## Materials and methods

### Study animals

All kinds of pigs in large-scale pig farms in Sichuan Province, including Large White, Landrace, Duroc, Pietrain, Landrace, Hampshire and Chenghua pigs, of all ages were included.

### Sample collection

A total of 174 stool samples and 160 bile samples were collected from 45 large-scale pig farms in various cities of Sichuan Province in China and maintained at −80 °C before performing experiments.

### HEV RNA extract and preparation of cDNA

Total RNA was extracted from HEV-positive material according to the Trizol method. Approximately 40 µl of RNase-free water was added to the total RNA, the pellet was gently pipetted with a pipette to dissolve it, and the sample was stored at −20 °C for later use. The extracted RNA was then reverse transcribed into cDNA using the Prime Script RT reagent kit. The volume of the transcription system was 10 µl. The reverse transcription conditions were as follows: 42 °C for 40 min and 85 °C for 5 min. The product was stored at −20 °C for later use.

### HEV RNA detection and sequencing

Two primers were used for the outer and inner PCR. The outer primers were HEV-F-Outer (5′-GCCCAGTATCGTGTTGTYC-3′) and HEV-R-Outer (5′-TARTCARCGGTATCCTCCAAA-3′), and the nested or inner primers were HEV-F-Inner (5′-CTGGYGTCGYTGAGGAAG-3′) and HEV-R-Inner (5′-CAGTRAGYGAAAGCCAAAGC-3′). The primers were commercially synthesised (IDT) and were used to amplify the targeted HEV ORF2 region.

The obtained cDNA in section ‘HEV RNA extract and preparation of cDNA’ was pre-denatured at 94 °C for 5 min, which was followed by 35 cycles of amplification at 94 °C for 40 s, 53 °C for 40 s and 72 °C for 1 min with a final extension at 72 °C for 10 min. The amplification product of the real-time PCR (RT-PCR) was used as a template for nested PCR using the inner primers. Both the reaction mixture volume and the PCR conditions used for the nested PCR were the same as those of the first round described above. The amplified nested PCR products were analysed using gel electrophoresis and visualised using Gel Doc (Bio-Rad), with a final amplification product of 638 bp.

### Sequence analysis

A total of 20 strains were selected from GenBank (refer to the virus information in Table 1). MegAlign software in the DNAStar package was used to clone the HEV ORF2 partial nucleic acid sequence of these clones, and the deduced amino acid sequences were compared with the corresponding sequences of 20 HEV reference strains. Molecular evolution analysis and homology comparison were carried out using the Kimura 2 parameter mode in the Neighbour-Joining algorithm (neighbour-joining method) of MEGA6.0 software with 1000 replicates from the set value to establish 20 sequencing strains and 20 reference strains for the genetic evolution of the tree, and the results were analysed and discussed. The nucleotide and deduced amino acid sequences of 13 sequenced genes were compared with the ORF2 partial gene sequences of the Chinese reference strain (GenBank accession number: DQ279091) using MEGA 5.0 (MegAlign) to analyse the amino acid and nucleotide mutations in the sequenced strains.

**Table 1.** Collected information on HEV reference strains and isolated strains with corresponding GenBank accession numbers

Accession	Isolate	Country	Genotype
AB220979	HE-JA41	Japan	IV
AB291968	JMM-Aba06C	Japan	IV
AB301710	JE03-1760F	Japan	III
AB573435	JBOAR135-Shiz09	Japan	V
EU360977	swX07-E1	Sweden	III
FJ705359	wbGER27	Germany	III
FJ763142	KNIH-hHEV4	South Korea	IV
GU206559	bjsw1	China	IV
JF443721	IND-HEV-AVH5-2010	India	I
JF443726	IND-HEV-FHF5-2007	India	I
JQ655734	W2-1	China	I
JQ655736	W2-5	China	IV
JQ953666	FR-SHEV3f	France	III
JQ993308	HEV-ZJ1	China	IV
KC692453	CH-YT-sHEV01	China	IV
KP294371	MWP-2010	Germany	III
KT447528	Sing-HEV23	Singapore	III
KU356186	Goat-HEV-52	China	IV
LC126332	P10_pJE03-1760F_wt	Japan	III
LC131066	huJB-0414	Japan	III
LC314155	HE-JA14-2173	Japan	I
M74506	/	Mexico	II
NC_001434	/	China	I

### Data analysis

Statistical analysis to determine the proportion of swine HEV (SHEV) infections within the study was conducted using Statistical Package for Social Sciences, version 22.0 (SPSS, Inc., Chicago, IL, USA). Sequence analysis and phylogeny was performed using Molecular Evolutionary Genetic Analysis (MEGA6) software.

## Results

### Identification of positive plasmid pMD19-T-SHEV

**Fig. 1.**
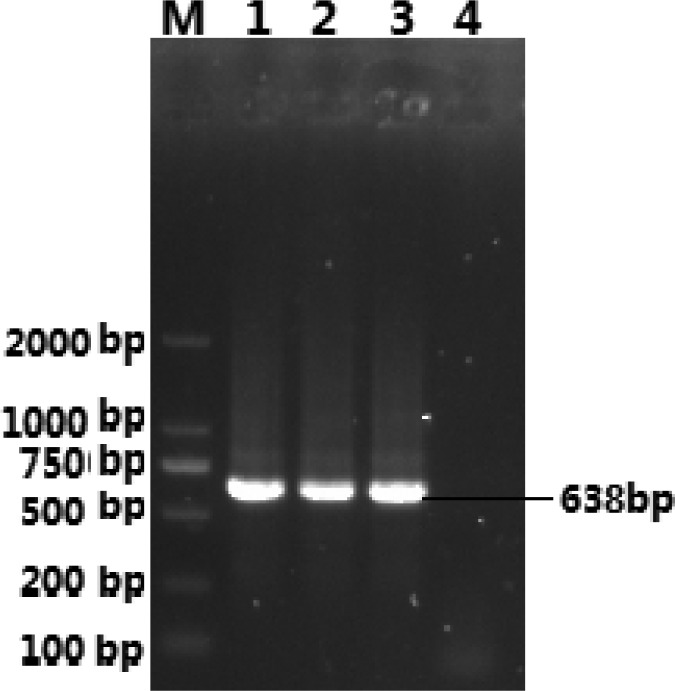
Identification of positive plasmids. M. DL2000DNA Marker; (1–3) pMD19-T-SHEV; (4) negative control.

The target fragment was ligated to the vector (Simple), and the ligation product was transformed into DH5*α* competent cells. The positive plasmid and no-load were identified by nested RT-PCR. The result showed that the size of the positive plasmid was the same as expected, indicating that the fragment was successfully cloned into pMD19-T vector (Fig. 1).

### SHEV infection in different areas of Sichuan Province

From August 2016 to October 2017, a total of 334 samples were collected from 45 pig farms in 12 cities in Sichuan Province using nested RT-PCR. The number of SHEV infections detected in each area, the number of swine farms tested and the corresponding positive results are shown in Table 2. The test results showed that SHEV was endemic in some areas; the positive rate of pig farms was 11.1% (5/45), and the total positive rate was 10.5% (35/334). The farms with the highest positive rates in Sichuan were in Suining. We did not detect SHEV in Dazhou, Guangyuan, Leshan, Luzhou, Yibin, Zigong or Ziyang. In addition, we found SHEV in other cities in Sichuan Province. The city with the highest positive sample rate was Chengdu. The 334 samples were divided into groups according to the age of the collected swine (4, 5–9 and 10 weeks of age). The SHEV infection statistics in swine of different ages are shown in Table 3. The age with the highest positive rate was 5–9 weeks at 17.9% (20/112), followed by pigs over 10 weeks old at 7.5% (5/67) and 1–4 weeks old at 6.5% (10/155). The HEV infection statistics in swine of different species are shown in Table 4. The examined swine species in order of highest to lowest SHEV infection rates were Chenghua pig, Large White, Duroc, Pietrain, Landrace and Hampshire.

**Table 2.** Detection of SHEV in different regions of Sichuan Province

City	Positive farms/total	Positive rate (%)	Positive samples/total	Positive rate (%)
Chengdu	2/10	20.0	15/54	27.8
Dazhou	0/2	0.0	0/20	0.0
Guangyuan	0/1	0.0	0/5	0.0
Leshan	0/3	0.0	0/22	0.0
Luzhou	0/4	0.0	0/35	0.0
Meishan	1/5	20	3/40	7.5
Mianyang	1/6	16.7	10/47	21.3
Nanchong	0/3	0.0	3/24	12.5
Suining	1/4	25	4/33	12.1
Yibin	0/2	0.0	0/12	0.0
Zigong	0/3	0.0	0/26	0.0
Ziyang	0/2	0.0	0/16	0.0
Total	5/45	11.1	35/334	10.5

**Table 3.** HEV infection rates by swine age

Age	Number of samples	Positive samples	Positive rate (%)
1–4 weeks	155	10	6.5
5–9 weeks	112	20	17.9
>10 weeks	67	5	7.5
Total	334	35	10.5

**Table 4.** HEV infection rates by swine species

Species	Number of samples	Positive samples	Positive rate (%)
Duroc	79	10	12.7
Large White	56	9	16.1
Landrace	90	6	6.7
Pietrain	63	5	7.9
Hampshire	36	2	5.6
Chenghua pig	10	3	30.0
Total	334	35	10.5

### Sequencing of amplified products and analysis

The 13 positive recombinant plasmids were sent to Bioengineering (Dalian) Co., Ltd., for sequence analysis. The returned sequences were analysed by BLAST software at NCBI to determine the HEV gene sequences. The sequences were named SHEV-CHN-SC01~SHEV-CHN-SC13. The sequences were sequenced and uploaded into GenBank (Accession No. MG866004–MG866016).

**Fig. 2.**
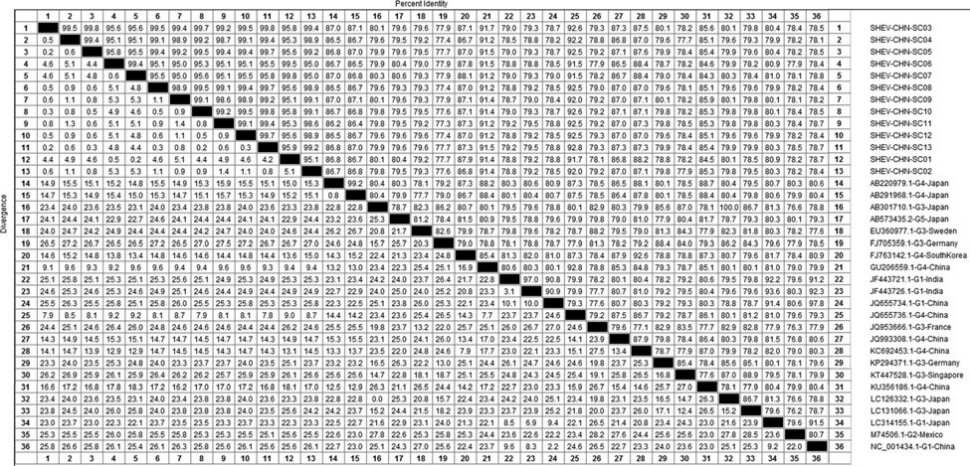
Analysis of nucleotide homologies for HEV ORF2 partial gene.

**Fig. 3.**
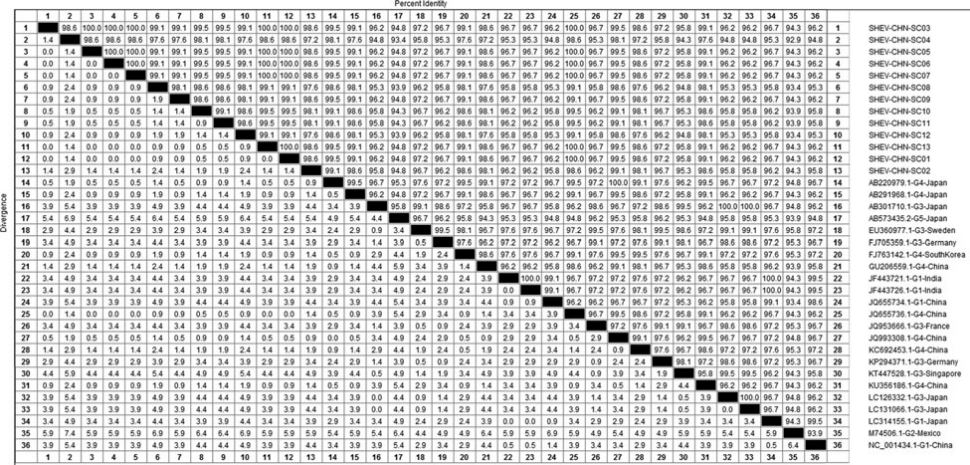
Analysis of amino acid sequence homologies for HEV ORF2 partial gene.

DNAStar software was used to analyse the nucleotide sequences and deduced amino acid sequences of the 20 HEV reference sequences published in this experiment. The results showed that the highest homology for the HEV ORF2 partial nucleotide sequence was found between the Sichuan sequences and Beijing, China, genotype IV (GenBank accession number: JQ655736) at 91.5–92.8% and that the lowest homology was found between the Sichuan sequences and Germany genotype III (GenBank accession number: FJ705359) at 77.3–77.9%. The highest homology of the HEV ORF2 partial amino acid sequence was found between the Sichuan sequences and Beijing, China, genotype IV (GenBank accession number: JQ655736) at 98.6–100.0% and that the lowest homology was found between the Sichuan sequences and Mexico genotype II (GenBank accession number: M74506) at 92.9–94.3% (Figs 2 and 3).

**Fig. 4.**
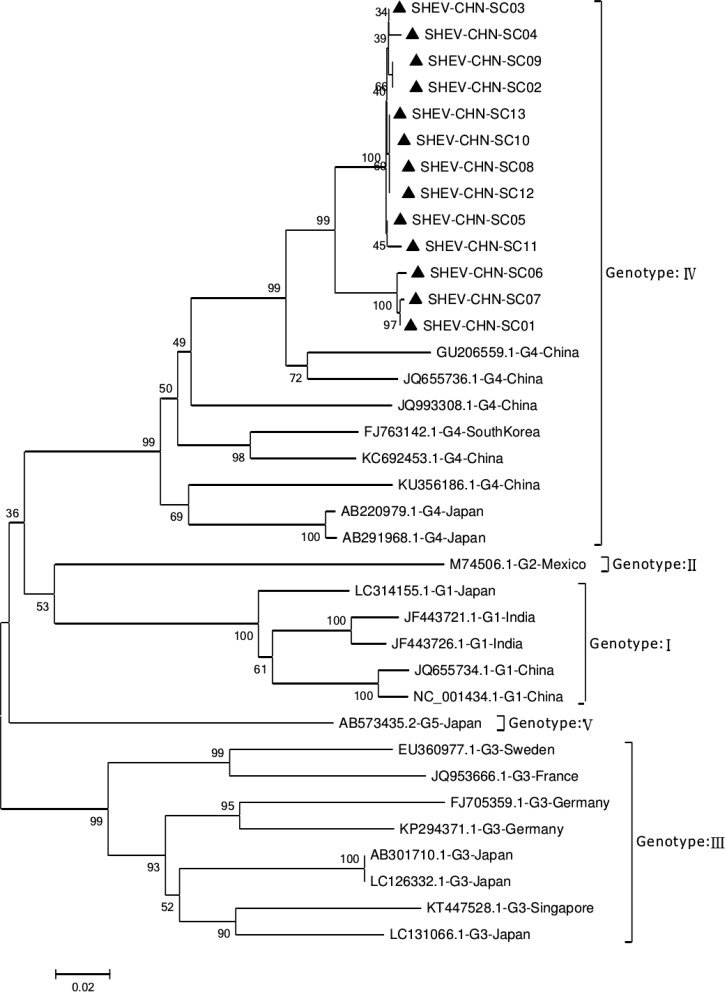
Evolutionary relatedness of our Sichuan isolates (black triangles) with different genotype HEV sequences inferred by molecular phylogenetic analysis for the HEV ORF2 partial gene. The evolutionary diversities were computed via the Kimura 2-parameter based on the number of substitutions per site. The variation among sites was modelled by a *γ* distribution. The tree is drawn to scale 0.1 with the branch length measured in the number of substitutions per site. The black triangles indicate Sichuan isolates belonging to genotype IV.

Phylogenetic analysis of the 13 SHEV ORF2 partial sequences amplified by DNAStar software and the 20 reference sequences downloaded from GenBank domestically and internationally was carried out. The data from some SHEV ORF2 sequences from 2016 to 2017 in select cities of Sichuan Province were used as the SHEV epidemic strains for a genetic evolution relationship diagram (Fig. 4). A phylogenetic tree based on the ORF2 partial sequence showed that the SHEV sequencing results were closest to sequence of the known Chinese strain but that the SHEV strains and the known Chinese strain were located on different branches; the SHEV strains grouped with the Korean strain.

**Fig. 5.**
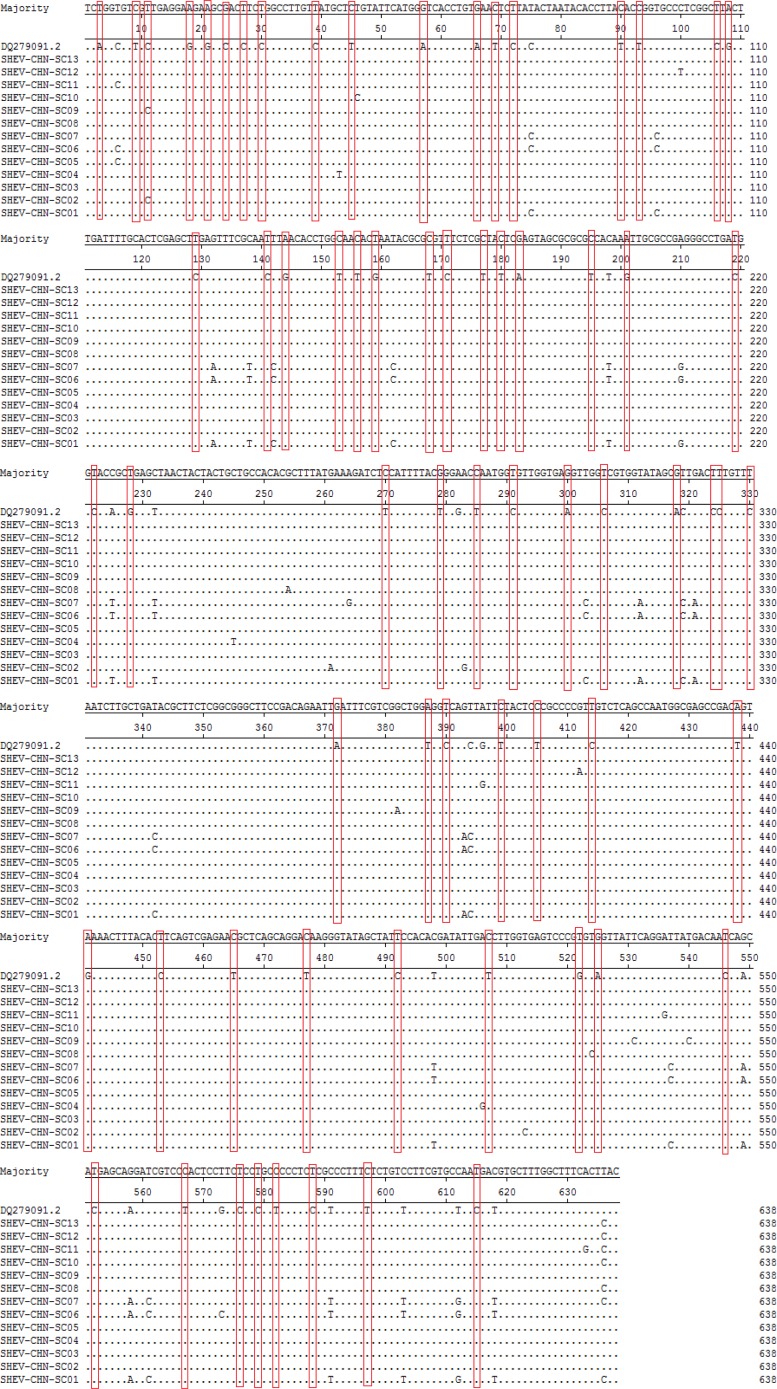
Deduced nucleotide alignments of the Sichuan (SC) swine HEV isolates with a reference swine HEV sequence from Heilongjiang, China. The red boxes indicate sites of nucleotide differences shared by all Sichuan isolates with respect to the reference isolate.

**Fig. 6.**
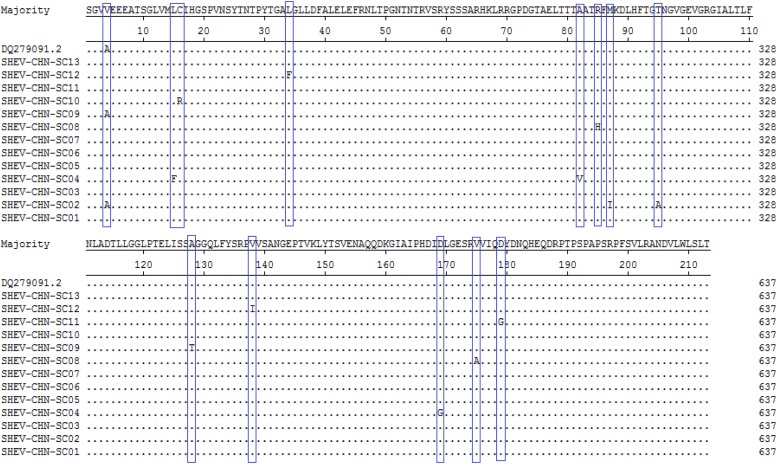
Deduced amino acid alignments of the Sichuan (SC) swine HEV isolates with a reference swine HEV sequence from Heilongjiang, China. The blue boxes indicate sites of amino acid differences shared by the Sichuan isolates with respect to the reference isolate, where F = phenylalanine, L = leucine, T = threonine, S = serine, G = glycine, A = alanine, V = valine, Q = glutamine, N = asparagine, P = proline, I = isoleucine, M = methionine, Y = tyrosine and D = aspartic acid.

A total of 115 nucleotide mutations were obtained by aligning the 13 sequences with the ORF2 gene of the Chinese reference strain (GenBank accession number: DQ279091) using MEGA 5.0 (MegAlign), and there were 68 common mutation sites in the 13 sequences and 47 different nucleotide mutation sites in total (Fig. 5). DNAMAN was used to predict the amino acid sequences of these 13 sequences. A total of 13 amino acid mutations were obtained by aligning the 13 sequences with the ORF2 gene of the Chinese reference strain (GenBank accession number: DQ279091) using MEGA 5.0 (MegAlign), and 13 different amino acid mutation sites were found in total, including variation sites at residues 4, 15, 16, 34, 82, 85, 87, 95, 128, 138, 169, 175 and 179 (Fig. 6).

## Discussion

HEV is a zoonotic disease that is prevalent in developing countries, such as Asian countries, African countries and Mexico. Swine infection with HEV is common in all farms worldwide. Studies have shown that pigs are the main storage hosts for HEV, and infected pigs generally have transient viremia for 1–2 weeks. The prevalence of HEV infection in Chinese farms is also very common. Researchers tested the serum of 8626 pigs in 120 large-scale pig farms in 20 provinces, municipalities and autonomous regions in China and found that the positive rate of anti-HEV antibodies was as high as 83.4%. HEV infection occurred at each of the investigated farms, indicating that HEV infection is an extremely serious issue for Chinese farms. Pigs that are naturally infected with HEV generally do not appear to have obvious clinical symptoms, so the establishment of a detection method with high sensitivity, easy operation and low costs is needed. At present, the established detection methods commonly use RT-PCR. However, RT-PCR detection has high costs, and it cannot be applied to large-scale clinical testing of pig farms. Therefore, this study established HEV nested RT-PCR, which is a simple procedure with high sensitivity and low costs.

In 2016, Liu *et al*. [17] investigated the HEV distribution in 15 provinces, autonomous regions and municipalities directly under the Central Government in China. However, the HEV distribution in some areas, such as Sichuan, Yunnan, Guizhou, Tibet and Chongqing, has not been investigated. To date, there have been no reports on the incidence of HEV in swine in Sichuan Province. In this study, primers were designed for the ORF region of HEV, and the nested RT-PCR method was used to analyse the incidence of HEV in Sichuan Province. Regional HEV prevention efforts provided data support. In this study, 334 pig manure and bile samples collected from large-scale pig farms in Chengdu, Mianyang, Meishan, Suining, Leshan and Yaan in Sichuan Province were tested; nine of these samples were positive, and the positive rate was 14.75%. The rates of SHEV infection were the highest in Chengdu and Mianyang at 27.8% and 21.3%, respectively. The RT-PCR results showed that 35 of the samples were positive and that the positive rate was 10.5%. Of the nested RT-PCR results, 35 samples were positive, and the positive rate was 10.5%. This study is the first time that HEV was detected in Sichuan Province, China. The coincidence rate of the positive results of the two methods was 100%. The results showed that the nested RT-PCR method established in this study had the same detection rate as RT-PCR. This study established the nested RT-PCR detection of HEV, and this method has the advantages of high sensitivity, specificity and quantification for clinical HEV diagnosis, representing a very good indicator for researchers in our country. The work lays the foundation for further study of HEV.

Chengdu, the area with the highest SHEV infection rate in Sichuan Province, may also be the first area infected with the disease, which serves as a reminder to farms in and around the area to focus on the environmental disinfection of pig farms to prevent the spread of HEV. The highest infection rate was found in 5–9-week-old swine, suggesting that farmers should pay attention to this age in prevention work. The species with the highest infection rate was the Chenghua pig, followed by the Large White Chenghua pig, Large White, Duroc, Pietrain, Landrace and Hampshire, all of which can be infected with SHEV; therefore, farms that breed these pigs should focus on preventing large-scale infection.

The ORF2 nucleotide sequence is the most stable sequence in the whole genome of HEV, and within this sequence, the section that overlaps with ORF3 is the most stable part of ORF2; however, it also encodes the most amino acid sequence variation [18]. The HEV ORF2 region encoding virus structural protein is the main focus of vaccine research. The results of nucleotide sequence analysis showed that there was high variation in the Sichuan SHEV sequences compared with the Chinese representative strain (GenBank accession number: DQ279091), suggesting that the first discovery of Sichuan SHEV was likely to be highly variant genotype IV HEV. The Sichuan epidemic strains were different from those endemic in other Chinese provinces and may also become new epidemic strains in the southwestern parts of China. Prevention and control work should be performed in all Chinese southwestern provinces and municipalities.

## Conclusion

This study revealed the prevalence of SHEV in different regions of Sichuan Province and found that the epidemic strain of SHEV in Sichuan was genotype IV. This study fills the gap in SHEV epidemiological research in Sichuan Province and lays the foundation for further research on HEV.
